# Supplementary material for: Species Identification and In Vitro Antifungal Susceptibility of Paecilomyces/Purpureocillium Species Isolated from Clinical Respiratory Samples: A Multicenter Study
Source: J Fungi (Basel). 2022 Jun 29;8(7):684. doi: 10.3390/jof8070684 (PMC9321559; doi:10.3390/jof8070684)
Supplement: Supplementary file 1 [file jof-08-00684-s001.zip › jof-1678510-supplementary.pdf]

Table S1. Concordance between MADLI-TOF and DNA gene sequencing identification.

DNA gene sequencing identification ITS	% of correct identification at species level by MALDI-TOF MS
Purpureocillium lilacinum (n=28)	100%
Paecilomyces variotii (n=26)	96%
Paecilomyces maximus (n=16)	94%

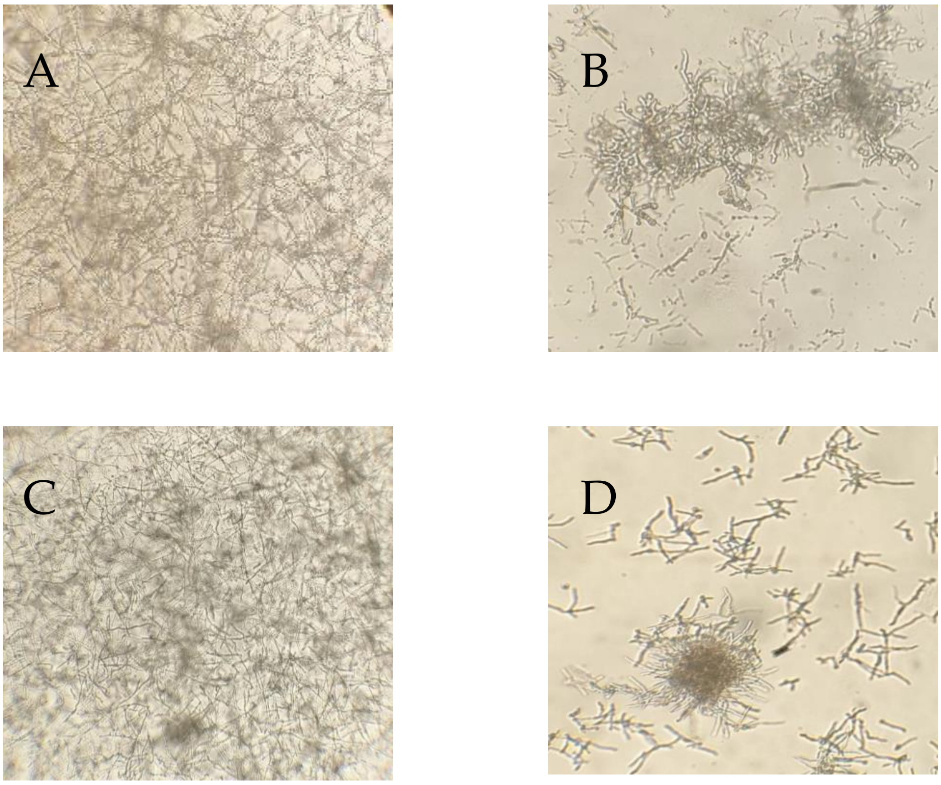

Figure S1. Fungal growth used for MECs determination. Magnification microscopy: 20x. **(A).** ***P.*** *variotii* (HEGP 5) control: uninhibited growth. **(B) Inhibition of** ***P.*** *variotii* (HEGP5) by 0.015 mg/l of anidulafungin (aberrant/blunted hyphal growth). **(C)** ***P.*** *lilacinum* (HEGP 20) control: uninhibited growth. **(D) Inhibition** ***P.*** *lilacinum* (HEGP 20) by 8 mg/l of anidulafungin (aberrant/blunted hyphal growth).
